# Case Report: Chanarin-Dorfman Syndrome: A Novel Homozygous Mutation in ABHD5 Gene in a Chinese Case and Genotype-Phenotype Correlation Analysis

**DOI:** 10.3389/fgene.2022.847321

**Published:** 2022-03-28

**Authors:** Bo Liang, He Huang, Jiaxiang Zhang, Gang Chen, Xiangsheng Kong, Mengting Zhu, Peiguang Wang, Lili Tang

**Affiliations:** ^1^ Department of Dermatology and Venereology, The First Affiliated Hospital, Anhui Medical University, Hefei, China; ^2^ Department of Clinical Laboratory, The First Affiliated Hospital, Anhui Medical University, Hefei, China; ^3^ Institute of Dermatology, Anhui Medical University, Hefei, China; ^4^ Key Laboratory of Dermatology (Anhui Medical University), Ministry of Education, Hefei, China; ^5^ State Key Laboratory Incubation Base of Dermatology, Anhui Medical University, Hefei, China; ^6^ Inflammation and Immune Mediated Diseases Laboratory of Anhui Province, Hefei, China; ^7^ Department of Occupational Health and Environment Health, School of Public Health, Anhui Medical University, Hefei, China; ^8^ Aberlong Biological Technology Co., Ltd., Shanghai, China; ^9^ Department of Clinical Medical, the First Clinical Medical College, Anhui Medical University, Hefei, China

**Keywords:** ichthyosis, ABHD5/CGI-58 gene, Chinese, Jordan’s anomaly, Chanarin-Dorfman syndrome

## Abstract

The Chanarin–Dorfman syndrome (CDS) is a rare, autosomal recessively inherited genetic disease, whch is associated with a decrease in the lipolysis activity in multiple tissue cells. The clinical phenotype involves multiple organs and systems, including liver, eyes, ears, skeletal muscle and central nervous system. Mutations in ABHD5/CGI58 gene have been confirmed to be associated with CDS. We performed whole exome sequencing on a Chinese CDS patient with skin ichthyosis features mimicking lamellar ichthyosis, ectropion, sensorineural hearing loss, and lipid storage in peripheral blood neutrophils. A novel homozygous missense mutation (p.L154R) in ABHD5 gene was detected in this patient. Genotype-phenotype analysis in reported CDS patients revealed no particular correlation. Our findings further enrich the reservoir of ABHD5 mutations in CDS.

## Introduction

Chanarin-Dorfman syndrome (CDS; OMIM 275630) is an extremely rare, multisystemic, autosomal recessive neutral lipid storage disorder (NLSD) arising from impaired lipid metabolism (Demerjian et al., 2006). CDS is associated with a multitude of clinical symptoms, the most prominent of which is icthyosis, especially non-bullous congenital ichthyosiform erythroderma. Patients can be born as collodion babies, occasionally accompanied by bilateral ectropion and eclabion. Other manifestations include liver steatosis, myopathy, sensoryneural hearing loss, and cataract (Yamaguchi and Osumi, 2009). To date, approximately 120 cases of CDS have been reported around the world, but mainly in Mediterranean and Middle Eastern countries, especially in Turkey (Incecik et al., 2018; Eskiocak et al., 2019; Louhichi et al., 2019; Niculescu et al., 2019; Al-Hage et al., 2020; Dabas et al., 2020; Cakmak and Bagci, 2021; Jiang et al., 2021; Tavian et al., 2021). So far, only three patients of CDS have been reported from China (Takeichi et al., 2016; Jiang et al., 2021).

CDS is caused by mutations of the abhydrolase domain containing 5 gene (*ABHD5*)/comparative gene identification-58 (*CGI-58*) on chromosome 3p21, leading to insufficient fatty acids (FAs) mobilization within the cell and systemic triglyceride accumulation in cytosolic droplets in multiple tissues. These lipid droplets have been observed in hepatocytes, intestinal mucosa, blood, bone marrow, skin fibroblasts, myocytes, central nervous system cells and many other types of cells.

The diagnosis is based on the presence of ichthyosis and identification of lipid droplets in granulocytes (Jordan’s anomaly) in peripheral blood smear. For patients with CDS, dietary modification has been reported to be an effective treatment, with no deleterious effects on liver function (Kakourou et al., 1997). Herein, we present a Chinese patient with CDS caused by a novel homozygous missense mutation, p. L154R, in *ABHD5* gene, and the genotype-phenotype correlation analysis was also conducted.

## Materials and Methods

### Case Report

**FIGURE 1:**
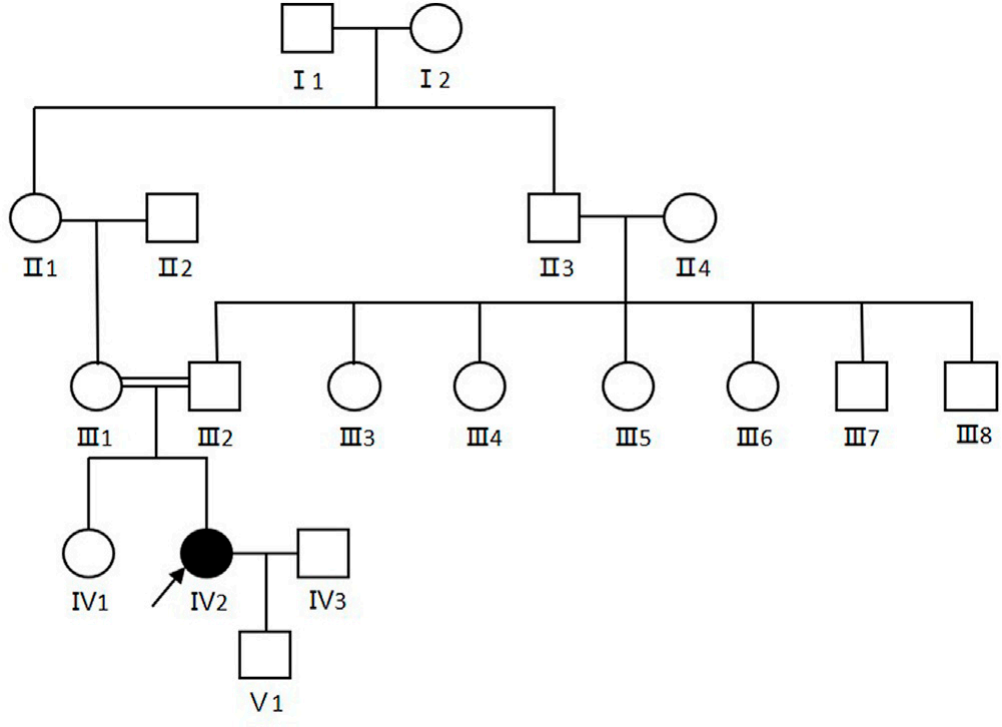
Pedigree of the CDS family. Arrows show the proband. Females were indicated by circles while males were indicated by squares. Blackened symbols represented patients who were carried the mutation through mutation sequencing.

The proband (Ⅳ2) in this study was a 30-year-old female displaying diffuse erythema, fine scaling on the body, and sensorineural hearing loss since her birth as a collodion baby. The severity of the ichthyosiform erythroderma had lessened as she aged. The condition was severe in winter and mild in summer. Her nails, teeth and hair appeared normal. No additional involvement of muscular system and central nervous system was found. Her parents were consanguineous (first cousins), and there were no other affected family members. Her son was 1 year old and was normal (Figure 1).

**FIGURE 2:**
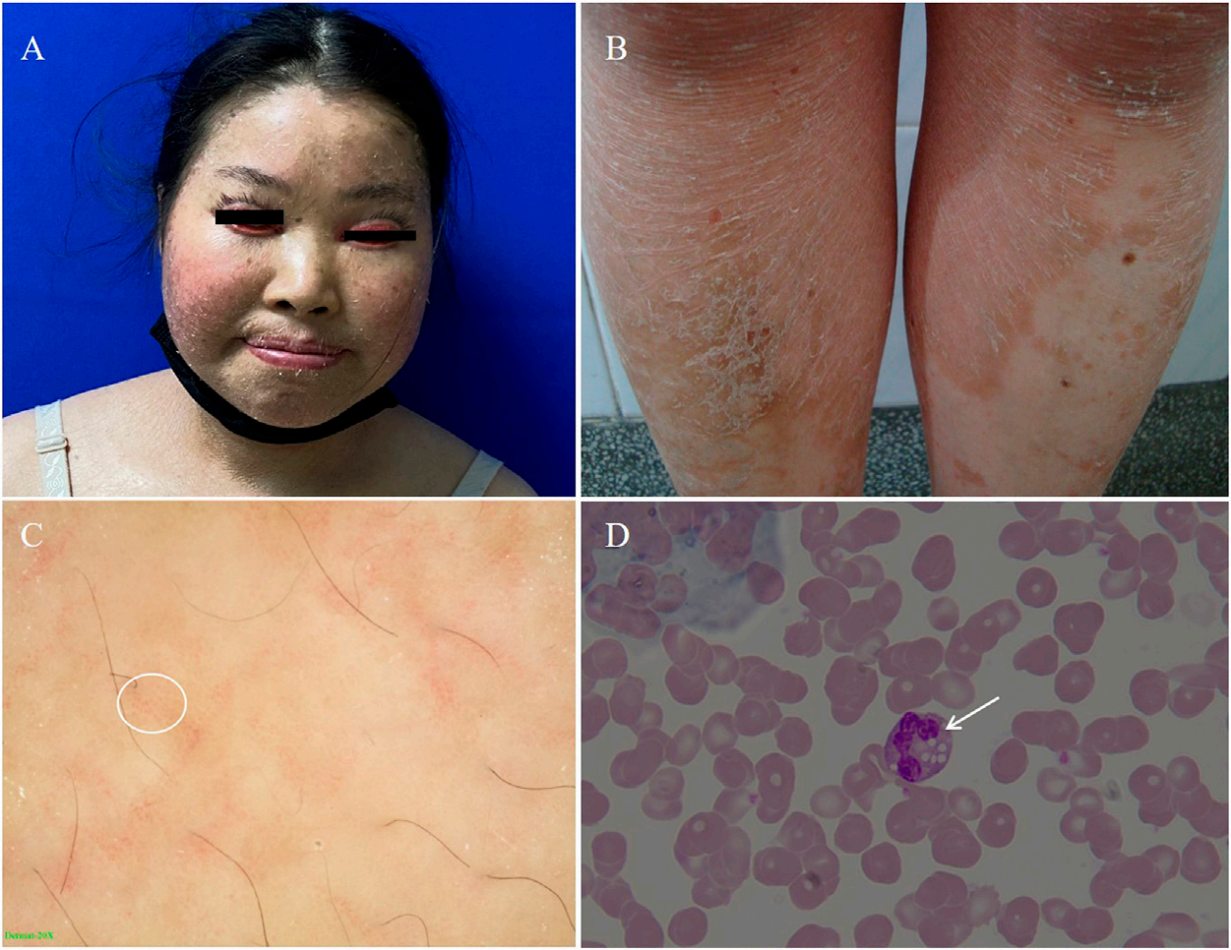
**(A)** Coarse facies of the proband with ptosis, bilateral extropion of the eyelids, broad forehead, depressed nasal bridge. **(B)** Nonbullous ichthyosiform erythroderma fine scales on the trunk. **(C)** The dermatoscopic appearance of the lesion on the trunk white scales and the diffuse, punctate haemorrhage of apparent blood capillaries (white circle as shown, 20×). **(D)** Peripheral blood smear. The arrow shows lipid vacuolization in leukocytes observed in blood smear (Jordan’s Anomaly) (Wright’s stain, 100×).

Physical examinations of the proband revealed coarse facial features, including ptosis, bilateral extropion of the eyelids, broad forehead, depressed nasal bridge, and extensive erythematous patch and plaques accompanied by fine scaling covering the body (Figures 2A,B). Dermatoscopy showed dilation of twisted capillaries and diffused white scales (Figure 2C). Laboratory findings revealed high levels of alanine aminotransferase (ALT, 108 U/L; normal 7–40 U/L), aspartate aminotransferase (AST, 87 U/L; normal 13–35 U/L), creatine kinase (CK, 400 U/L; normal 100–250 U/L), and low levels of urea (2.42 mmol/L; normal 2.60–7.50 mmol/L), and vitamins D (28 ng/ml; normal 30–100 ng/ml). Triglycerides and total cholesterol levels were normal. Abdominal ultrasound showed moderate fatty infiltration of the liver without splenomegaly. Test of the peripheral blood revealed distinct lipid accumulation in polymorphonuclear cells (Jordan’s anomaly, Figure 2D).

### Peripheral Blood Collection and DNA Extraction

After obtaining informed consent from all participants and approval from Clinical Research Ethics Committee of Anhui Medical University, EDTA anticoagulated venous blood samples were collected from the family. Genomic DNA was extracted using a Flexi Gene DNA Kit (250) in a standard procedure and stored at −80°C. The procedures were in accordance with the Helsinki Declaration of 1975, as amended in 1983.

### Whole Exome Sequencing

WES was performed in the proband (IV2). Genomic DNA fragments corresponding to all exons in genome were amplified by PCR and subjected to automatic DNA sequencing after purification. Agilent SureSelect XT Library Prep Kit and SureSelect Human All Exon V6 kit were used for the library preparation and capture. Illumina Hiseq XTen platform was used for the sequencing. Screening for disease-associated deleterious mutations was made with emphases on all the possible pathogenic variations in reported *ABHD5* gene.

### Sanger Sequencing

The possible pathogenic variations identified by WES were confirmed by Sanger sequencing in the proband’s father (Ⅲ2) and sister (IV1) to detect genotype-phenotype co-segregation. Primers flanking all coding regions of the possible variation were designed using software Primer Premier 5.0 (Primer Biosystems, Foster City, CA, United States). PCR products from genomic DNA were sequenced using an ABI 3730XL DNA Analyzer (ABI, Foster City, CA, United States). The sequencing results were analyzed using Finch TV (Version 1.5), and the newly discovered mutation was named referring to the principle of the Human Genome Variation Society (HGVs).

### Review of the Literature

Articles published between 1974 and 2021 were searched on PubMed by using the following keywords singly or in various combinations: “Chanarin–Dorfman Syndrome”, “Dorfman–Chanarin syndrome”, “congenital ichthyosiform erythroderma”, “neutral lipid storage disorder”, “*ABHD5*/*CGI-58* mutation” and “Jordan’s anomaly”. The patients’ race, age, gender, clinical symptoms, genetic mutations were all evaluated.

## Results

### WES Results and Co-Segregation Analysis

**FIGURE 3:**
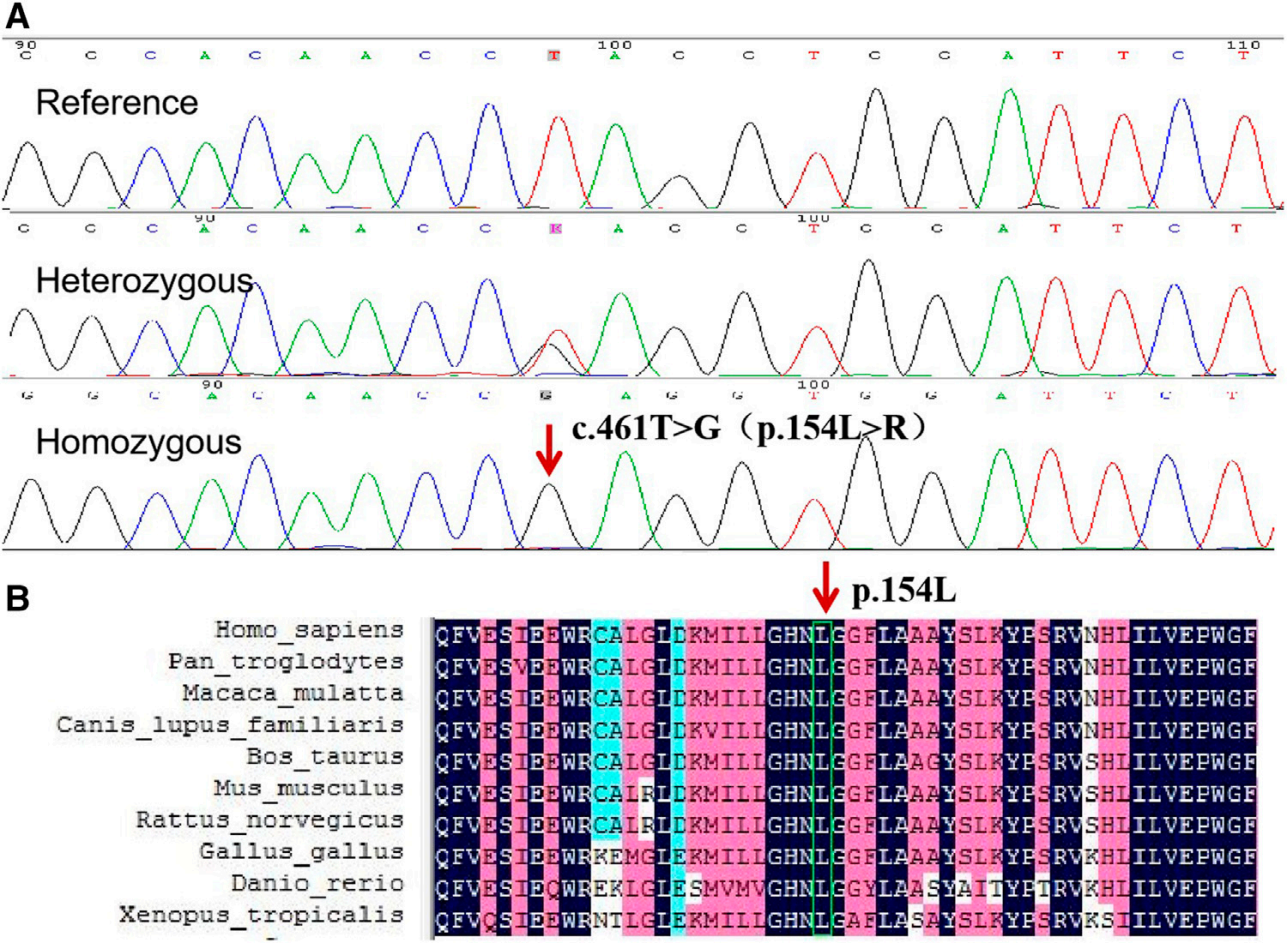
**(A)** Sequence analysis of *ABHD5* gene in the proband and her sister. The arrow indicates the homozygous mutation c.461T > G, which results in the protein change, p. L154R. **(B)** Conservation analysis using DNAMAN revealed that ABHD5 amino acid sequence at position 154 are highly conserved across multiple species.

WES revealed a novel homozygous missense mutation c.461T > G (NM_016,006) in *ABHD5* gene, resulting in the substitution of amino acid arginine for leucine at position 154 (p.L154R), which is a highly conserved amino acid leucine across multiple species (Figures 3A,B). The mutation was predicted by REVEL to be pathogenic and by SIFT to be damaging, with scores of 0.959 and 0, respectively. Sanger sequencing revealed the mutation was homozygous in the proband and heterozygous in her father and sister. (Figure 3A). The mutation was not found in 100 control individuals from the same ethnicity, and was not recorded in the database of genomAD.

### Genotype-Phenotype Correlation Analysis

We found 106 CDS patients (58 male) reported in literature in whom the molecular analysis of *ABHD5* gene were performed (Supplementary Table S1). The age of the patients varied from 4 months to 67 years. A total of 45 mutations in *ABHD5* have been identified, including 37 homozygous mutations and 8 compound heterozygous mutations (Supplementary Table S1). The mutations identified in the patients included missense, nonsense, insertions, deletions, and frameshift mutations. Irrespective the nature of the mutation, all CDS patients showed the typical skin features of non-bullous congenital ichthyosiform erytroderma and Jordan’s anomaly, followed by hepatomegaly and hepatosteatosis. The most common mutation in patients is p. N209X (26/45, 57.8%). Within the group of N209X mutation patients, the CDS phenotype was homogeneous. This mutation was identified in 26 cases (23 from Turkey), and is rare in other populations. The other mutations mostly appear to be familial or local. No particular genotype-phenotype correlation were found in the literature.

## Discussion

In the present study, the clinical features, laboratory findings and genetic results of the proband were consistent with the diagnosis of CDS. And mutation analysis of *ABHD5* using WES and Sanger sequencing revealed a new homozygous mutation. For this mutation, p. L154R, leucine is a hydrophobic amino acid, while arginine is an alkaline amino acid. The transition of the amino acid polarity may affect the structure and function of ABHD5 protein.

To date, 45 different mutations in the *ABHD5* gene have been reported (Incecik et al., 2018; Eskiocak et al., 2019; Louhichi et al., 2019; Niculescu et al., 2019; Al-Hage et al., 2020; Dabas et al., 2020; Cakmak and Bagci, 2021; Jiang et al., 2021; Tavian et al., 2021), among which the homozygous mutation of p. N209X is the most common. A comparison of findings in patients with the common N209X mutation and other mutations did not show major differences, and does not point to a particular genotype-phenotype correlation, which is consistent with previous researches (Aggarwal et al., 2012; Nur et al., 2015). The variability of clinical symptoms in patients with CDS depends on a large number of mutations involved, and the severity of the phenotype can be quite variable. Ichthyosis from birth was a universal presentation, followed by liver disease. It is reported that there was an intrafamilial phenotypic heterogeneity in the alive affected individuals, which led to the hypothesized that mutations in other genes might have affected the phenotypes through modifier effects (Takeichi et al., 2016). Furthermore, the lack of correlation between the genotype and the severity of the disease may be explained by the role of epigenetic and environmental factors. Liver involvement is an important cause of mortality and morbidity in CDS patients. Most of the patients with cirrhosis identified in the literature had advanced age (Cakmak and Bagci, 2021). However, it is reported that the cirrhosis may develop at an early age depending on the nature of the mutations (Aggarwal et al., 2012; Tamhankar et al., 2014). So, it is possible that there is some genotype-phenotype correlation. More CDS cases with mutation data are needed to confirm the genotype-phenotype correlations in *ABHD5* mutations.

Mutations in *ABHD5* may lead to the accumulation of long-chain fatty acids, energy deficiency in cells and affect the skin barrier (Tamhankar et al., 2014). The ABHD5 protein has been studied as a cofactor for adipose triglyceride lipase (ATGL). Ujihara er al. revealed that the triglycerides levels in the scales from the patient were positively correlated with the severity of ichthyosis (Ujihara et al., 2010). The level of triglyceride in this patient was normal, which may be a reason for the mild clinical manifestation of this patient. It has been shown that ABHD5 participates in the assembly and release of hepatitis C virus particles by mobilizing the lipid pool of cytoplasmic lipid droplets, therefore, CDS patients may show certain resistance to hepatitis C virus infection (Vieyres et al., 2016).

The diagnosis of CDS can be confirmed by performing a peripheral blood smear to show lipid vacuoles in granulocytes, myocytes, hepatocytes, fibroblasts and keratinocytes, a feature called Jordan’s anomaly. Ichthyosiform erythroderma, as a usual symptom of CDS, is a typical manifestation in congenital ichthyosis syndromes. Studies found that the co-existence of Jordan’s anomaly and ichthyosis provided the definitive diagnosis in CDS, regardless of the *ABHD5* gene mutation (Cakmak and Bagci, 2021). Therefore, PBS is necessary to examine for the presence of Jordan’s anomaly. In peripheral blood smears taken from our patients, lipid vacuolization was seen in the cytoplasm of leukocytes. This finding supports the presence of a natural lipid storage disorder.

There has been no curative treatment of CDS so far. Various topical therapies, including emollients and keratolytic agents, have been proposed to improve ichthyosis, with mostly unsatisfactory results. While systemic therapy with retinoids combined with dietary modification has been used successfully in patients with skin and muscle manifestations (Gandhi et al., 2007; Israeli et al., 2012), co-morbidities limit its use in CDS. Niculescu et al. proposed tazarotene 0.015% cream as a potential topical agent for patients with ichthyosis, including patients with systemic involvement (Niculescu et al., 2019). In the present study, a diet low in fat and rich in short/medium-chain fatty acids and emollients were administered. After 1 year of treatment, the skin lesions improved, and the patient was satisfied with the treatment effect and attended the scheduled follow-up visits.

In conclusion, the patient we presented showed ichthyosiform dermatosis, and mutation analysis eventually confirmed the diagnosis of CDS. CDS presents suffer from damage of many systems, and the severity of the phenotype can be quite variable. The diagnosis can be established by the clinical features and a blood smear, which can be confirmed by the molecular analysis. The mutation found in this patient enriched our understanding of pathogenic mutations for CDS. As it is not easy to obtain an accurate diagnosis only based on the dermal features, a blood smear and mutational analysis are required for patients suspected with congenital ichthyosis (Cheng et al., 2020).

## Data Availability

The datasets for this article are not publicly available due to concerns regarding participant/patient anonymity. Requests to access the datasets should be directed to the corresponding authors.
